# The relationship between quality of life, health and care transition: an empirical comparison in an older post-acute population

**DOI:** 10.1186/1477-7525-10-69

**Published:** 2012-06-15

**Authors:** Leah Couzner, Julie Ratcliffe, Maria Crotty

**Affiliations:** 1 Department of Rehabilitation and Aged Care, Flinders University, Adelaide, South Australia; 2 Flinders Centre for Clinical Change and Health Care Research, Flinders University, Adelaide, South Australia

**Keywords:** Aged, Geriatrics, Rehabilitation, Quality of life, Health services for the aged, Health economics

## Abstract

**Background:**

The aim of this study was to explore, via empirical comparison, the relationship between quality of life, as measured by the ICECAP-O capability index (a new instrument designed to measure and value quality of life in older people), with both self-reported health status and the quality of care transition in adults aged 65 and over participating in two post acute rehabilitation programs (outpatient day rehabilitation and the Australian National Transition Care residential program).

**Methods:**

The ICECAP-O was administered to patients receiving either outpatient day rehabilitation (n = 53) or residential transition care (n = 29) during a face to face interview. The relationships between the ICECAP-O and other instruments, including the EQ-5D (a self-reported measure of health status) and CTM-3 (a self-reported measure of the quality of care transitions), the type of post-acute care being received and socio-demographic characteristics were examined.

**Results:**

The mean ICECAP-O score for the total sample was 0.81 (SD: 0.15). Patients receiving outpatient day rehabilitation generally reported higher levels of capability, than patients receiving residential transition care (mean 0.82 [SD: 0.15] and 0.79 [SD: 0.164] respectively), however these differences were not statistically significant. The mean EQ-5D score for the total sample was somewhat lower than the ICECAP-O (mean 0.55; SD: 0.27) indicating significant levels of health impairment with the outpatient day rehabilitation group demonstrating slightly higher levels of health status than the transition care group (mean 0.54 [SD: 0.254] and mean 0.49 [SD: 0.30]). The ICECAP-O was found to be positively correlated with both the CTM-3 (Spearman’s r =0.234; p ≤ 0.05) and the EQ-5D (Spearman’s r = 0.437; p ≤ 0.001). The relationships between the total EQ-5D and CTM-3 scores and the individual attributes of the ICECAP-O indicate health status and quality of care transition in this patient population to be influential in some, but not all aspects of capability.

**Conclusions:**

The correlations between the ICECAP-O, EQ-5D and CTM-3 instruments illustrate that capability is strongly and positively associated with health-related quality of life and the quality of care transitions. However further research is required to further examine the construct validity of the ICECAP-O and to examine its potential for incorporation into economic evaluation.

## Background

The life expectancy of Australians is among the highest in the world and is expected to continue to increase, contributing to an ageing population [1]. Currently 13.5% of the total Australian population are aged 65 years and over, a number that is projected to increase to up to 25% by 2056 [2]. This is predicted to result in an increased demand for care and support services and the funding of new technologies, leading to a need for increased resource allocation in a variety of areas including hospitals, pharmaceuticals, medical benefits and private health insurance [3,4].

While the need for the Australian health system to provide services which are cost-effective has been widely acknowledged, ideally this should be undertaken in a way that does not minimise the quality of care provided to recipients. The escalating pressure faced by heath systems in relation to the allocation of scarce health care resources highlights the importance of economic evaluations of health and social care interventions in informing such decisions. Quality of life has been recognized as an important outcome of health and social care interventions, and as such is frequently measured in economic evaluations via the calculation of quality adjusted life years (QALYs) which combine health-related quality of life with the period of time spent in each health state. However some interventions may impact on quality of life more broadly than the health-related aspects encapsulated by the QALY. For example, rehabilitation interventions for older people following an acute hospital stay may include education, problem solving, therapy, medical interventions and the provision of aids, e.g. electric wheelchairs and walking aids, in order to promote independent living. Whilst the provision of these interventions may have little or no impact upon health, they may lead to significant improvements in an individual’s quality of life [5]. From a health economics perspective, it is therefore important to be able to measure and value quality of life in a way that is suitable for inclusion within an economic evaluation framework, yet which also encompasses the multitude factors that may influence quality of life.

Many countries are expanding the range of post-acute services available to older people partly in response to shortening hospital stays and partly in response to community demand for additional services to reduce the risk of institutionalisation and maintain older people independently in their own homes. The Australian National Transition Care Program was established in 2005 to reduce the length of inappropriate hospital stays and premature admission to residential aged care facilities. The program is targeted at older people at the end of an inpatient hospitalisation who were not eligible for hospital rehabilitation services but who required further care in order to complete their recovery, optimise their level of functioning and make arrangements for long term care if necessary. The program is time-limited and goal-oriented and can be provided in either an inpatient or community setting. The services provided are determined by individual need and can include low intensity rehabilitation, medication support, case management and nursing and personal care. Prior to commencing Transition Care, patients are required to be medically stable and approved for transition care by an Aged Care Assessment Team [6]. A recent evaluation of the Australian Transition Care program demonstrated transition care provided in the community could reduce both hospital readmissions and transfers to residential care settings [7,8].

This study sought to empirically compare the relationship between quality of life, self-reported health and the quality of care transition in adults aged 65 and over whom were receiving post acute care either by participating in outpatient day rehabilitation or receiving residential Transition Care utilising the newly developed ICECAP-O instrument.

## Method

Participants were recruited from an outpatient day rehabilitation unit at the Repatriation General Hospital, a 300 bed acute care hospital in metropolitan Adelaide, South Australia, and City Views a residential transitional care facility providing post-acute rehabilitation type care to adults aged 65 and over who require further recovery time to assess whether they could go home or required residential care admission These two patient groups were selected as together they are broadly representative of the post-acute population of older people in South Australia. The two groups represent a range of post acute patients’ levels of functional independence with the transition care group being functionally more dependent than the outpatient day rehabilitation group [8]. Participants were eligible for inclusion if they were currently receiving post-acute care in the form of either outpatient rehabilitation or the residential Australian National Transition Care Program, were within 3 months of an acute hospital admission, were aged 65 years or over and had a Mini Mental State Examination score of 24 or above [9]. The study sample was obtained sequentially over a 17 month period from August 2009 to January 2011. Admission lists were monitored weekly by research staff for new patients who met the study inclusion criteria. Eligible patients were approached while at either the outpatient day rehabilitation unit or the residential transitional care facility by the same research staff and provided with verbal and written information about the study. All patients who went on to participate in the study provided informed, written consent to do so. The study was approved by the Southern Adelaide Health Service / Flinders University Human Research Ethics Committee.

Data were collected via a face to face interview with individual participants. Participants were asked to complete the ICECAP-O, EQ-5D and CTM-3 instruments. Basic socio-demographic information was also collected such as age, gender, country of birth, level of education, diagnosis and residential status.

### Measures

#### ICECAP-O index of capability

The ICECAP-O index of capability is a newly developed instrument designed to measure and value quality of life, as defined by an individual’s capabilities, for application within economic evaluations of health and social care interventions. The instrument is designed for utilisation with older people (aged 65 years plus) and focuses on quality of life in a broader sense rather than health-related quality of life alone. This means that the ICECAP-O has the potential to inform resource allocation decision making across health, social and aged sectors, [10,11]. In a study to assess the construct validity of the instrument, Coast and colleagues [11] found that individuals’ quality of life was influenced by their capability to achieve the attributes included in the ICECAP-O instrument. The instrument is comprised of 5 attributes: attachment, role, enjoyment, security and control. The attributes were developed via qualitative interviews with older people about what was important to them in terms of their quality of life [10,11]. Each attribute contains five levels from which participants choose the level that best matches their current situation. A preference based scoring algorithm was developed for the instrument using a best-worst scaling discrete choice experiment (DCE) with a representative sample of older people in the United Kingdom [10]. The scoring algorithm can be readily applied to obtain a single index value for all possible combinations of responses ranging from 0 (no capability) to 1 (full capability). This facilitates the potential for the ICECAP-O to be used to measure and value the benefits of health, social and aged care interventions.

#### EQ-5D

The EQ-5D is a generic measure of health status which is widely applied in the economic evaluation of health care treatments and services. The instrument is comprised of a descriptive system covering five dimensions: mobility, self-care, usual activities, pain/discomfort and anxiety/depression, each consisting of 3 levels of increasing severity [12]. The EQ-5D enables the calculation of a single index value indicating quality of life ranging from 0 (worst imaginable health) to 1 (perfect health) which can be used in economic evaluations [12]. Indeed, the instrument has been widely applied in this context with older adult general populations in the community and older adult patient populations [13-15].

#### CTM-3

The Care Transition Measure was designed to measure the quality of transitions between health care settings from the older patient’s perspective. This can then be used in the evaluation of health service performance [16].The instrument covers 3 domains: whether the patient understood what they were responsible for in managing their health, the purpose of their medications and whether their preferences and those of their family were taken into consideration. Individual responses to the CTM-3 are used to calculate a score ranging from 0 to 100, with higher scores indicating higher quality transitions [17]. The instrument has been used internationally by organisations involved in health care delivery, quality improvement and research [17].

Basic descriptive tests were used to provide a summary of respondent’s characteristics. The data were analysed using SPSS version 17. Spearman’s rho was used to examine the association between continuous variables and chi-squared tests, analysis of covariance and T-Tests were performed to assess the associations between categorical variables.

A priori hypotheses were formed about the expected relationships between the ICECAP-O and the other measurement tools and socio-demographic data based upon previous assessments of validity for other measures and evidence of relationships from previously published studies (where available), and the views of the research team members where other evidence was not available.

##### Health

It was anticipated that there would be a strong relationship between health status and capability, supporting previously published studies presented by the developers of the ICECAP-O [11]. It was therefore hypothesised that participants reporting high levels of capability would also experience high levels of self-reported health. The ICECAP-O attributes of control, enjoyment and role were expected to be associated with health status as they are more closely related to physical health than the remaining 2 domains. It was also hypothesized that each of the EQ-5D domains, would exhibit a strongly associated with capability, but particularly mobility, self-care and usual activities.

##### Quality of care transitions

Previous studies have indicated that there may be a positive relationship between the quality of care transitions and health related quality of life [8,18]. Given the strong relationship between health status and capability previously identified, it was therefore anticipated that there may also be a positive relationship between the quality of care transitions and capability with those individuals who scored more highly on the CTM-3 exhibiting higher levels of capability according to the ICECAP-O.

##### Socio-demographic characteristics

A relationship was expected between capability and the type of post-acute care being received. It was anticipated that participants receiving outpatient rehabilitation would have higher scores on average than the participants receiving the Australian National Transition Care Program as they had returned to the community to live post-acutely while the Transition Care recipients were still requiring institutional care and assistance with activities of daily living.

## Results

### Participant Characteristics

Of the 96 eligible patients approached for participation in the study, 86 (90%) agreed to participate. A total of 4 participants had incomplete ICECAP-O data due to a refusal to answer particular ICECAP-O questions or were unable to fully complete the interview giving a total useable response rate of 85% (82/96). Fifty-three of these participants were receiving outpatient rehabilitation, while the remaining 29 were receiving residential transition care. The characteristics of the participants who completed the ICECAP-O instrument are presented in Table 1. The participants had a mean age of 76 years (SD 7.4) and were evenly split in regards to gender (female: 50%, n = 41). At the time of interview, the participants had been receiving post-acute care for a mean duration of 37 days (SD 18.4). Neurological diagnoses were the most common reasons for receiving post-acute care (n = 30, 37%) followed by orthopaedic diagnoses (n = 26, 32%). The majority of participants were not living alone prior to their acute hospital admission (57%, n = 47) and reported having an informal carer (65%, n = 56).

**Table 1:** Characteristics of participants (n = 82)

***Characteristic***	***Outpatient Rehab (n = 53)***	***Transition Care (n = 29)***	***Total (n = 82)***
Age (mean, SD)	74.87 (7.17)	80.69 (6.27)	76.23 (7.38)
Cognitive score (mean, SD)	27.83 (1.23)	28.17 (1.49)	27.95 (1.78)
Gender: Female (%)	24 (45)	17 (59)	41 (50)
Has a carer (%)	33 (60)	21 (72)	54 (67)
***Residential status (%)***			
Living alone	14 (26)	21 (72)	35 (43)
Living with others	39 (74)	8 (28)	47 (57)
***Country of birth (%)***			
Australia	40 (76)	24 (83)	64 (78)
Other	13 (25)	5 (17)	18 (22)
***Highest education***			
No education	1 (2)	-	1 (1)
Primary or secondary	24 (45)	19 (66)	43 (52)
Tertiary	28 (53)	8 (28)	36 (44)
Post-acute care duration (mean, SD)	33.21 (16.03)	42.59 (21.11)	36.52 (18.42)
***Reason for post-acute care (%)***			
Neurological	29 (55)	1 (3)	30 (37)
Orthopaedic	8 (15)	18 (62)	26 (32)
Functional decline/falls/mobility	10 (19)	6 (21)	16 (20)
Other	6 (11)	4 (14)	10 (12)
***Instrument scores***			
ICECAP-O (mean, SD)	0.82 (0.15)	0.79 (0.16)	0.81 (0.15)
EQ-5D (mean, SD)	0.54 (0.25)	0.49 (0.30)	0.52 (0.27)
CTM-3 (mean, SD)*	75.79 (16.78)	63.60 (17.67)	71.48 (17.97)

* Significant difference between sites at between the 1% and 10% levels.

The mean ICECAP-O score for the total sample was 0.81 (SD 0.15). As predicted, the transition care group demonstrated lower levels of capability (mean 0.79, SD 0.16) than the rehabilitation group (mean 0.82, SD 0.15), although not to a statistically significant level. The distribution of responses to the ICECAP-O from participants who answered all five questions is presented in Table 2. The participants reported high levels of attachment (56%, n = 48) and security (38%, n = 31). Limitations at various levels were evident however in terms of role (78%, n = 64), enjoyment (70%, n = 57) and control (87%, n = 71). A participant was deemed to have a limitation in a particular capability if they selected the second, third or fourth level for that particular question of the ICECAP-O instrument.

**Table 2:** Distribution of responses to the ICECAP-O instrument (n = 82)

	***Outpatient Rehab (n = 53)***	***Transition Care (n = 29)***	***Total (n = 82)***
Attachment			
I can have all of the love and friendship that I want	34 (64.2%)	13 (44.8%)	47 (57.3%)
I can have some of the love and friendship that I want	13 (24.5%)	12 (41.8%)	25 (30.5%)
I can have a little of the love and friendship that I want	5 (9.4%)	4 (13.8%)	9 (11.0%)
I cannot have any of the love and friendship that I want	1 (1.9%)	-	1 (1.2%)
Security			
I can think about the future without any concern	17 (32.1%)	14 (48.3%)	31 (37.8%)
I can think about the future with only a little concern	21 (39.6%)	8 (27.6)	29 (35.4%)
I can only think about the future with some concern	9 (17.0%)	7 (24.1%)	16 (19.5%)
I can only think about the future with a lot of concern	6 (11.3%)	-	6 (7.3%)
Role			
I am able to do all of the things that make me feel valued	13 (24.5%)	7 (24.1%)	20 (24.4%)
I am able to do many of the things that make me feel valued	20 (37.4%)	8 (27.6%)	28 (34.2%)
I am able to do a few of the things that make me feel valued	15 (28.3%)	9 (31.0%)	24 (29.3%)
I am unable to do any of the things that make me feel valued	5 (9.43%)	5 (17.2%)	10 (12.2%)
Enjoyment			
I can have all of the enjoyment and pleasure that I want	18 (34.00%)	9 (31.0%)	27 (32.9%)
I can have a lot of the enjoyment and pleasure that I want	22 (41.5%)	9 (31.0%)	31 (37.8%)
I can have a little of the enjoyment and pleasure that I want	12 (22.6%)	9 (31.0%)	21 (25.6%)
I cannot have any of the enjoyment and pleasure that I want	1 (1.9%)	2 (6.9%)	3 (3.7%)
Control			
I am able to be completely independent	13 (24.5%)	2 (6.9%)	15 (18.3%)
I am able to be independent in many things	25 (47.2%)	16 (55.2%)	41 (50.0%)
I am able to be independent in a few things	13 (24.5%)	9 (31.0%)	22 (26.8%)
I am unable to be at all independent	2 (3.8%)	2 (6.9%)	4 (4.9%)

### Self-Reported Health Status

The participants who completed the ICECAP-O instrument had a mean EQ-5D score of 0.52 (SD 0.27), with the rehabilitation recipients exhibiting higher scores than the transition care recipients (mean 0.54, SD 0.25 and mean 0.49, SD 0.30 respectively) as hypothesised, although not to a statistically significant level (Table 1). It was anticipated a priori that participants reporting high levels of capability via the ICECAP-O would also report high levels of self-rated health as measured by the EQ-5D. The distribution of mean EQ-5D values across the ICECAP-O levels of capabilities is presented in Table 3. This data is based upon participants who answered all five of the questions in the ICECAP-O instrument. A linear increase in EQ-5D scores was accompanied by increases in all of the ICECAP-O domains except attachment and security; however an upward trend was evident for these attributes.

**Table 3:** Distribution of mean EQ-5D values across ICECAP-O levels of capabilities (n = 82)

***Attribute***	***Outpatient Rehab (n = 53)***	***Transition Care (n = 29)***	***Total (n = 82)***
Attachment			
I can have all of the love and friendship that I want	0.568	0.490	0.545
I can have a lot of the love and friendship that I want	0.569	0.498	0.441
I can have a little of the love and friendship that I want	0.398	0.493	0.472
I cannot have any of the love and friendship that I want	0.088	-	0.088
Security			
I can think about the future without any concern	0.565	0.565	0.565
I can think about the future with only a little concern	0.607	0.444	0.562
I can only think about the future with some concern	0.430	0.408	0.420
I can only think about the future with a lot of concern	0.425	-	0.425
Role			
I am able to do all of the things that make me feel valued	0.504	0.748	0.599
I am able to do many of the things that make me feel valued	0.648	0.355	0.564
I am able to do a few of the things that make me feel valued	0.484	0.493	0.487
I am unable to do any of the things that make me feel valued	0.371	0.361	0.366
Enjoyment			
I can have all of the enjoyment and pleasure that I want	0.555	0.721	0.615
I can have a lot of the enjoyment and pleasure that I want	0.623	0.431	0.567
I can have a little of the enjoyment and pleasure that I want	0.394	0.389	0.392
I cannot have any of the enjoyment and pleasure that I want	0.312	0.225	0.254
Control			
I am able to be completely independent	0.595	0.872	0.634
I am able to be independent in many things	0.578	0.549	0.567
I am able to be independent in a few things	0.441	0.379	0.416
I am unable to be at all independent	0.312	0.190	0.230

The ICECAP-O was found to be positively associated with the EQ-5D (Spearman’s r = 0.426; p ≤ 0.001), as shown in Table 4, indicating that an increase in capability were accompanied by an increase in self-reported health status. This is further supported by the significant associations between the ICECAP-O and EQ-5D scores shown in Table 5 (p ≤ 0.001), suggesting a relationship between capability and self-reported health. As shown in Table 6, a significant positive association was evident between the ED-5D scores and the ICECAP-O domain of control (p<0.05), indicating that some, but not all, aspects of capability are influenced by self-reported health.

**Table 4:** Relationship between the ICECAP-O, EQ-5D and CTM-3 calculated using Spearman’s rho (n = 82)

	***ICECAP-O***
EQ-5D^**a**^	0.437**
CTM-3	0.234*

^a^ n = 80 due to incomplete EQ-5D data.

** correlation is significant at the 1% level or higher.

* correlation is significant at between the 1% and 10% levels.

**Table 5:** Tests of association (P values) between the ICECAP-O tariff and other characteristics measured using T-Tests and ANOVA (n = 82)

***Characteristic***	***P Value***
Site	0.429
Age	0.614
Gender	0.083
Cognitive status	0.280
Residential status	0.501
Has a carer	0.258
Country of birth	0.316
Education^**a**^ (n = 80)	0.259
Post-acute care duration	0.826
Reason for post-acute care	0.509
EQ-5D: mobility	0.028*
EQ-5D: self-care	0.007*
EQ-5D: usual activities	≤0.001**
EQ-5D: pain/discomfort	0.995
EQ-5D: anxiety/depression	0.041*
EQ-5D: overall value	≤0.001**
CTM3: hospital staff	0.981
CTM3: managing health	0.173
CTM3: purpose of medications	0.111
CTM-3: overall value	0.161

^a^ n = 80 due to missing education data.

** Association is significant at the 1% level or higher.

* Association is significant at between the 1% and 10% level.

**Table 6:** Tests of association (P values) between capabilities as measured by the ICECAP-O and other characteristics using chi-squared tests (n = 82)

	***Attachment***	***Security***	***Role***	***Enjoyment***	***Control***
Site	0.279	0.119	0.670	0.495	0.251
Age	0.580	0.117	0.660	0.975	0.864
Gender	0.760	0.474	0.799	0.274	0.332
Cognitive status	0.631	0.232	0.106	0.480	0.545
Residential status	0.019*	0.552	0.540	0.232	0.614
Has a carer	0.474	0.890	0.007*	0.525	0.934
Country of birth	0.232	0.111	0.047*	0.105	0.207
Education^a^	0.656	0.916	0.142	0.785	0.196
Post-acute care duration	0.858	0.656	0.623	0.645	0.449
Reason for post-acute care	0.042*	0.633	0.670	0.898	0.875
EQ-5D: mobility	0.335	0.105	0.771	≤0.001**	≤0.001**
EQ-5D: self-care	0.721	0.317	0.018*	0.122	0.003*
EQ-5D: usual activities	0.704	0.668	≤0.001**	0.058	0.004*
EQ-5D: pain/discomfort	0.069	0.073	0.616	0.081	0.140
EQ-5D: anxiety/depression	0.279	0.419	0.364	0.339	0.023*
EQ-5D: overall value^a^	0.741^a^	0.088^a^	0.092^a^	0.058^a^	0.043^a^ *
CTM3: hospital staff	0.947	0.761	0.459	0.956	0.499
CTM3: managing health	0.020*	0.912	0.097	0.191	0.510
CTM3: purpose of medications	0.810	0.468	0.169	0.125	0.177
CTM-3: overall value	0.139	0.712	0.035*	0.145	0.015*

^a^ n = 80.

** Association is significant at the 1% level or higher.

* Association is significant at between the 1% and 10% level.

### Quality of care transitions

Table 7 presents the distribution of responses to the CTM-3 from participants who completed all five questions of the ICECAP-O instrument. The mean CTM-3 score for the total sample was 71.48 (SD 17.97). The mean score of the rehabilitation patients (mean 75.79, SD 16.78) was higher than that of the transition care recipients (mean 63.60, SD 17.67) to a level that was statistically significant (p<0.05). The CTM-3 was positively correlated with the ICECAP-O (Spearman’s r =0.217; p ≤ 0.05) (Table 4). This suggests that higher quality care transitions are accompanied by higher levels of capability in support of our prior hypothesis. Although no significant relationship was found between the ICECAP-O and CTM-3 scores (Table 5), a significant relationship was found to exist between the CTM-3 score and the ICECAP-O attributes of role and control, indicating that some, but not all areas of capability are influenced by the quality of care transitions (Table 6).

**Table 7:** Distribution of responses to the CTM-3 (n = 82)

	***Outpatient Rehab (n = 53)***	***Transition Care (n = 29)***	***Total (n = 82)***
The hospital staff took my preferences and those of my family or caregiver into account in deciding what my health care needs would be when I left the hospital			
Strongly disagree	1 (1.9%)	1 (3.4%)	2 (2.4%)
Disagree	8 (15.1)	7 (24.1%)	15 (18.3%)
Agree	26 (49.1%)	12 (41.4%)	38 (46.3%)
Strongly agree	14 (26.4%)	3 (10.3%)	17 (20.7%)
Don’t know/not applicable	4 (7.5%)	6 (20.7%)	10 (12.2%)
When I left the hospital I had a good understanding of the things I was responsible for in managing my health			
Strongly disagree	-	2 (6.9%)	2 (2.4%)
Disagree	5 (9.4%)	5 (17.2%)	10 (12.2%)
Agree	22 (41.5%)	15 (51.7%)	37 (45.1%)
Strongly agree	25 (47.2%)	4 (13.8%)	29 (35.4%)
Don’t know/not applicable	1 (1.9%)	3 (10.3%)	4 (4.9%)
When I left the hospital I clearly understood the purpose for taking each of my medications			
Strongly disagree	-	2 (6.9%)	2 (2.4%)
Disagree	6 (11.3%)	3 (10.3%)	9 (11.0%)
Agree	20 (37.7%)	13 (44.8%)	33 (40.2%)
Strongly agree	27 (50.9%)	11 (37.9%)	38 (46.3%)
Don’t know/not applicable	-	-	-

### Socio-demographic characteristics

Tests of association between ICECAP-O scores and socio-demographic characteristics revealed no significant relationships, as shown in Table 5. Despite a priori expectation, no relationship was found to exist between age and any of the ICECAP-O attributes. Of significance however was the relationship between the ICECAP role attribute and country of birth (p<0.05) and whether or not the participant had an informal carer (p<0.05) as shown in Table 6. A relationship was also evident between the ICECAP attachment attribute and residential status (p<0.05) (Table 6). Participants who reported having an informal carer were more likely to experience role limitations.

## Discussion

The findings of this empirical comparison suggest the existence of a strong relationship between the concepts of capability, self-reported health and the quality of care transitions when measured in a post-acute setting (transition care or outpatient rehabilitation) using the ICECAP-O, EQ-5D and CTM-3 instruments. The capability of this population was slightly lower than that reported in other studies utilising the ICECAP-O instrument [10,19]. However this may be attributable to the previous studies being based upon samples of the United Kingdom general population, both of which were younger in age than the current sample. The participants in this study who reported high levels of care transition quality also displayed higher capability levels. The quality of care transitions experienced by the participants was similar to that recorded in other studies of similar populations [8,17]. Higher levels of capability were also evident in the participants exhibiting higher levels of self-reported health. Although participants in this study demonstrated lower levels of self-rated health than in another study of older adults [11], our participants were older and recovering from an acute hospitalisation. The associations between self-reported health and the capability domains suggest health status to be influential in some, but not all aspects of capability, echoing the findings of previous work [11] which also revealed strong, positive relationships between self-reported health and some, but not all capabilities as measured using the ICECAP-O instrument.

The absence of a relationship between capability and socio-demographic characteristics is indicative that, in this population, self-reported health and the quality of care transitions were more influential than socio-demographic factors on capability. This is in contrast to the findings of Coast and colleagues [11] who found a strong association between capability and age. However those findings were based upon members of the United Kingdom general population, while this study focused on older Australian sample who recovering from a recent acute illness.

The relatively small sample size is a limitation of this study. We achieved a high consent rate of 93% and the sample contained a diverse range of diagnoses broadly representative of older people attending outpatient rehabilitation and transition care programmes. However, it is important that further research is conducted to verify these preliminary findings in larger clinical samples. In addition, as no Australian alternatives were available at the time the study was conducted, the ICECAP-O and EQ-5D scoring algorithms that were applied were the original algorithms for each instrument which are based on the values of the UK general population. However, Flynn and colleagues [20] are currently in the process of developing a scoring algorithm for the ICECAP-O instrument based upon the preferences of the Australian general population and an Australian general population scoring algorithm has recently been developed for the EQ-5D [21]. Further studies conducted in Australian patient and general population samples should apply the new Australian general population algorithms pertaining to each instrument.

The data presented here were collected as part of a wider study focusing on the application of a discrete choice experiment to elicit the preferences of patients participating in either outpatient day rehabilitation or receiving residential transition care. Further measurement of capability at multiple time points would be beneficial in establishing the re-test reliability of the ICECAP-O and its sensitivity to change over time. Further research should also be conducted to compare the ICECAP-O with other instruments designed to measure quality of life more broadly amongst older people e.g. the recently developed OPQOL (Older People’s Quality of Life) instrument [22,23]

The use of the ICECAP-O capability index provides an alternative approach for to the measurement and valuation of the quality of life of older people. The ICECAP-O focuses on quality of life more broadly, rather than concentrating on health alone, and has the potential to be applied to aid in the determination of resource allocation decisions across the health, social and aged care sectors. In this study, utilisation of the ICECAP-O has provided insight into the relationship between capability, self-reported health and the quality of care transition in a post-acute population. However future research is required to further examine the construct validity of the ICECAP-O and its potential for application within economic evaluation in larger clinical settings and in alternative settings and populations of older adults.

## Competing interests

The authors have no competing interests to declare

## Authors’ contributions

LC collected the participant data and drafted the manuscript. JR participated in the study design and drafted the manuscript. MC participated in the study design. All authors read and approved the final manuscript.
